# The multifaceted role of ASICs channels into the nervous system and their potential use as a therapeutic target for neuro-disorders

**DOI:** 10.1007/s00702-026-03165-5

**Published:** 2026-05-05

**Authors:** Nunzia Maisto, Sepideh Dashtiani, Asia D’Ettorre, Silvia di Nuzzo, Dalila Mango

**Affiliations:** 1 School of Pharmacy, Department of Biology, University of Rome Tor Vergata, 00133 Rome, Italy; 2 Department of Physiology and Pharmacology “V. Erspamer”, Sapienza University of Rome, 00185 Rome, Italy; 3 Department of Biomedicine and Prevention, University of Rome Tor Vergata, 00133 Rome, Italy

**Keywords:** Acid-sensing ion channels, Glia cells, Neurodegeneration, Neuropharmacology, Neuroinflammation, Synaptic plasticity

## Abstract

Acid-sensing ion channels (ASICs) are increasingly recognized as promising therapeutic targets within both the central and peripheral nervous systems, with significant relevance to neurodegenerative and neuroinflammatory pathologies. Among these, ASIC1a has been most closely implicated in the progression of neurodegeneration, primarily through its roles in mediating excitotoxicity, intracellular Ca²⁺ overload, and pro-inflammatory signalling cascades. Pathological acidification, a common feature of many neurological disorders, activates ASICs, contributing to neuronal injury and the activation of glial cells, which together sustain a deleterious cycle of inflammation and neurodegeneration. Importantly, ASICs are not confined to neurons but are also expressed in glial populations, including astrocytes and microglia, where their activation by extracellular acidosis can initiate and amplify immune responses. This glial involvement positions ASICs as modulators of neuroimmune dynamics, suggesting that their inhibition could mitigate both neuronal and glial-mediated pathology. A growing body of preclinical evidence supports the therapeutic potential of ASICs inhibition, with several pharmacological antagonists demonstrating the capacity to preserve synaptic integrity, attenuate glial activation, and reduce neuronal loss. Targeting ASICs thus offers a dual-pronged strategy that addresses both neuronal damage and glial-driven inflammation. This review aims to examine the roles of ASICs in both neuronal and glial cells across the CNS and PNS, and to evaluate whether modulation of ASICs activity may represent a strategy to move beyond symptomatic relief toward disease-modifying therapies that target the underlying mechanisms of neurodegenerative disorders.

## Introduction

Neurodegenerative diseases pose a major global health challenge, as current therapeutic strategies primarily aim to alleviate symptoms rather than prevent or reverse disease progression. These disorders are characterized by progressive neuronal dysfunctions, synaptic loss, and chronic neuroinflammation, ultimately leading to cognitive impairment and motor deficits (Rao et al. 2012; Agnello and Ciaccio 2022; Shaw et al. 2023). In addition, they are frequently associated with a neuroinflammatory state, which contributes significantly to neurological morbidity, often exacerbating neurodegeneration through persistent immune activation and demyelination (Rao et al. 2012; De Araújo Boleti et al. 2020; Shaw et al. 2023). Given the complex and multifactorial nature of these disorders, there is an urgent need to identify novel molecular targets that can modulate disease pathophysiology rather than merely manage symptoms.

One emerging therapeutic target is the acid-sensing ion channel (ASICs) family, a group of proton-gated ion channels widely expressed throughout the central (CNS) and peripheral nervous system (PNS), whose activation by extracellular acidification has been implicated in neuronal dysfunction and injury under pathological conditions (Wemmie et al. 2013; Deval and Lingueglia 2015; Boscardin et al. 2016). Interestingly, ASICs inhibition in different preclinical models of neurodegeneration demonstrated a significant reduction in neuronal death, inflammation and synaptic dysfunction (Xiong et al. 2004; Friese et al. 2007; Kreple et al. 2014; Mango et al. 2017; Mango and Nisticò 2019; Ribeiro Liberato et al. 2024), making it a compelling neuroprotective approach.

This review provides a comprehensive exploration of the pivotal role of ASICs in neurodegenerative and neuroinflammatory disease, shedding light on their role in physiological processes, thereby highlighting their contributions to disease progression and neuronal dysfunction, concepts already well-established and herein updated with recent findings. By examining the potential of inhibition as an innovative therapeutic strategy, this review underscores how these proton-gated ion channels serve as key modulators of neuropathological processes in both CNS and PNS, highlighting their role also in the glial cells. Targeting ASICs represents a promising frontier in neuropharmacology, offering new avenues for disease-modifying treatments that go beyond symptomatic relief.

## Structure and functions of ASICs: physiological and pathological considerations in both the central and peripheral nervous systems

ASICs are family epithelial sodium channel/degenerin (ENaC/DEG) members, widely expressed in the nervous system. The endogenous ligand is the proton (H^+^), and activation of the ligand-gated ion channel allows Na^+^ and Ca^2+^ to pass through the pore into the cell.

### Structure and functions of ASICs

**Fig. 1:**
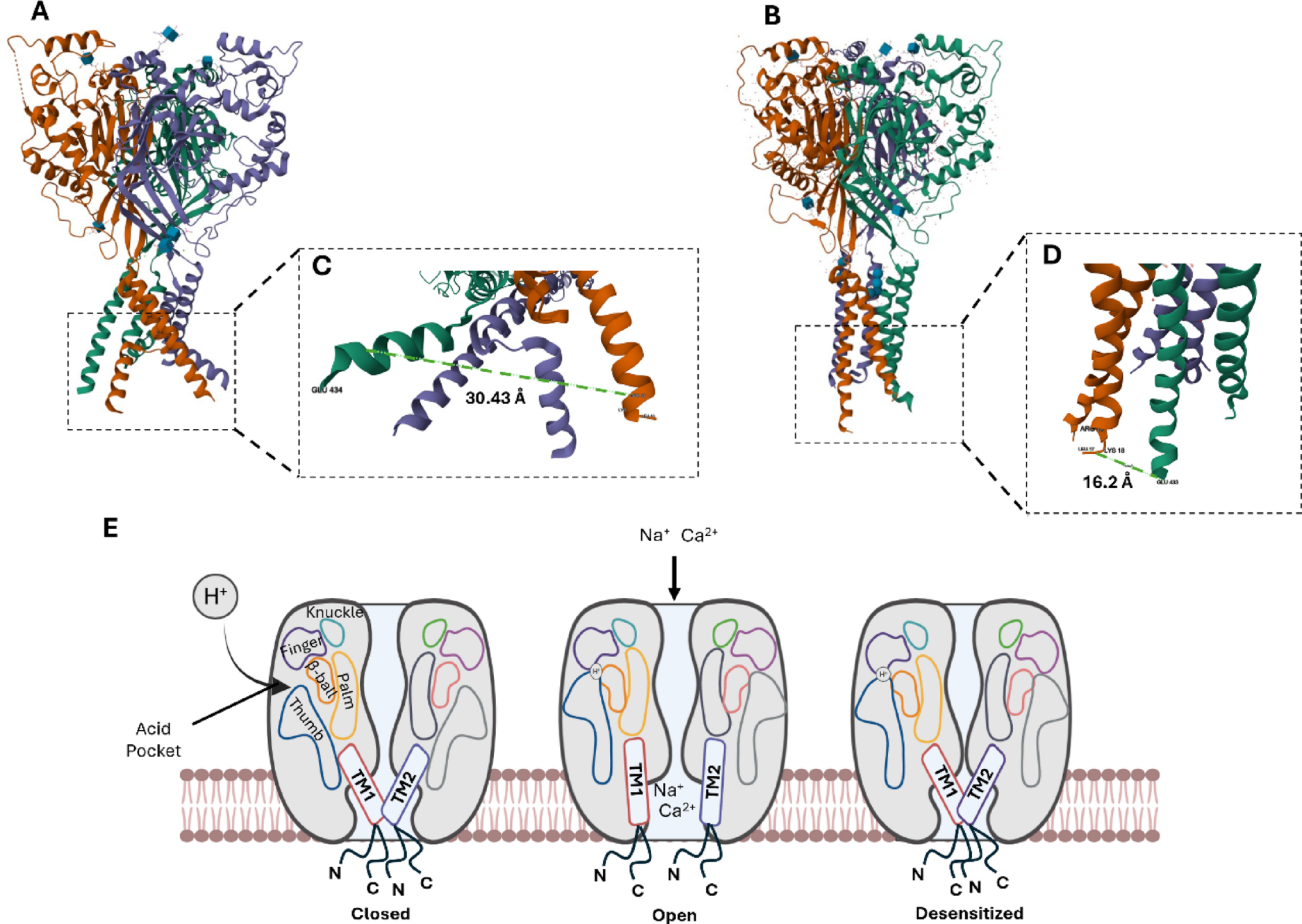
Structural and functional transitions of acid-sensing ion channels 1a (ASIC1a). **A** Cryo-EM structure of ASIC1a in the closed (resting) state, showing relaxed extracellular domains and tightly packed TM2 helices occluding the pore (PDB 5WKU). **B** Structure in the desensitised state, highlighting the conformational tightening of the acidic pocket and a collapsed, non-conductive pore (PDB 2QTS). **C-D** Zoomed structures to highlight the gating transitions. Insects show key distances between residues (30.43 Å vs. 16.2 Å) reflecting conformational rearrangements. **E** Schematic representation of the ASIC1a subunits and gating cycle: at resting pH, the channel is closed; extracellular acidification promotes proton binding, loops compression, and pore opening via TM2 splaying; prolonged acid exposure leads to desensitisation, with the pore re-closing despite maintained acidic conditions. Each subunit contributes two transmembrane domains (TM1 and TM2), a large extracellular loop, and intracellular termini, forming a trimeric channel complex with diverse functional properties. (Created by Biorender.com)

There are different subtypes of ASICs, which are determined by their main subunits (ASIC1a, ASIC1b, ASIC2a, ASIC2b, ASIC3, ASIC4) (Waldmann et al. 1997; Wemmie et al. 2006, 2013). While ASIC1a, ASIC2a, and ASIC2b are expressed in both the CNS and PNS, ASIC1b and ASIC3 are primarily localised to the PNS (with low levels of ASIC3 also detected in the human CNS), ASIC1a is by far the predominant subtype in the human and rodent brain (Wemmie et al. 2013; Wu et al. 2016). Each subunit contains two transmembrane domains, a large extracellular domain enriched in acidic residues, and intracellular N- and C-terminal domains (Fig. 1). A functional ASICs channel is composed of three subunits, assembling into either homo- or hetero- trimeric complexes (Jasti et al. 2007a; Gautam and Benson 2013; Bartoi et al. 2014), determinising their unique biophysical properties, including pH sensitivity, activation and inactivation kinetics, ion selectivity, and pharmacological profiles (Fig. 1) (Wemmie et al. 2002; Hesselager et al. 2004; Sherwood et al. 2011, 2012; Rook et al. 2021). In the closed (resting) state, the acidic pocket is relaxed, and the ion pore is sealed by inward-facing TM2 helices. Upon extracellular acidification, proton binding induces conformational tightening of the extracellular domain, causing the TM2 helices to splay outward and open the pore (open state) (Fig. 1). This acidification occurs in vivo under particular circumstances, such as inflammation, ischemia, tissue injury, or high synaptic activity, during which the extracellular pH can decrease transiently or persistently, reaching values low enough to activate ASICs, typically between pH 7.0 and as low as 6.0, or even lower during pathological states. This pH range determines the physiological vs. pathological activation of ASICs, thereby relating the molecular activation of ASICs to their biological context. When the low pH is sustained, the channel transitions to a desensitised state: while the acidic pocket remains compressed, the pore undergoes a secondary rearrangement and re-collapses into a non-conductive configuration, leading to the channel inactivation (Fig. 1) (Baconguis et al. 2014; Vullo et al. 2017; Yoder et al. 2018; Rook et al. 2020; Yoder and Gouaux 2020). Additionally, variations in subunit composition influence the channels’ response to extracellular acidosis and their modulation by endogenous factors and pharmacological agents, highlighting their diverse functional roles in neural signalling and plasticity characterized by a pH transient shift (Jasti et al. 2007b; Gründer 2020). Under sufficiently strong or prolonged extracellular acidification, which exceeds the range of physiological pH fluctuations, ASICs can adopt multiple desensitized states whose kinetics and stability depend on both the magnitude and duration of the pH change (Zhang et al. 2023; Holm et al. 2024). Among these, ASIC1a is particularly notable for its high H^+^ sensitivity and unique ability to activate neurotoxic signalling pathways in response to pathological acidosis (Xiong et al. 2004). Unlike other subtypes, ASIC1a rapidly and completely desensitises (Salinas et al. 2009; Wang et al. 2015; Boscardin et al. 2016). Interestingly, Ca²⁺ influx, previously considered the primary mechanism of acid-toxic neuronal death, does not fully account for the prolonged neurodegeneration observed in sustained acidosis (Mari et al. 2010; Wang et al. 2015). Recent studies, indeed, reveal that ASIC1a can trigger necrotic cell death, independently of ion conduction through its interaction with receptor-interacting threonine/serine protein kinase 1 (RIPK1) (Wang et al. 2024). This interaction initiates necroptosis-mediated cell death by activating RIPK1 phosphorylation, even in the absence of ion flux through the channels (Wang et al. 2015, 2024). This discovery challenges the classical view of ASIC-mediated neuronal injury as purely Ca^2+^ dependent, highlighting broader signalling capabilities of ASIC1a beyond ion permeability.

ASICs participate in neurophysiological and neuropathological processes (fear, learning, anxiety, substance abuse, and neurodegeneration) through their ion-conducting activity, which modulates neurotransmission upon activation by H^+^. Specifically, ASIC activation leads to Na⁺ influx, while ASIC1a channels additionally allow Ca²⁺ entry, resulting in neuronal depolarization and activation of voltage-gated Ca²⁺ channels and N-methyl-D-aspartate receptors (NMDARs) (Gao et al. 2005, 2015), with the consequent Ca²⁺/calmodulin-dependent protein kinase II (CaMKII) phosphorylation, which regulates signalling pathways involved in synaptic plasticity, learning, memory, and pain processing (Gao et al. 2005, 2015; Wemmie et al. 2013). To further support their role in neurotransmission, recent research has uncovered a novel aspect of ASIC1a modulation, highlighting more of the ASIC1a-NMDAR interplay: glutamate, a classical neurotransmitter, acts as a positive allosteric modulator of ASIC1a (Lai et al. 2024a). This binding enhances ASIC1a activity, potentiating H^+^-induced currents (Lai et al. 2024a), and it may exacerbate Ca²⁺ influx contributing to mitochondrial dysregulation and neurotoxicity (Savic Azoulay et al. 2020), especially in neurodegenerative conditions. Certainly, ASICs are integrated in mitochondrial membranes, highlighting a pivotal role in regulating Ca^2+^ homeostasis, essential for cellular energy production (Wang et al. 2013; Liu et al. 2018; Savic Azoulay et al. 2020; Noterman et al. 2021). This interplay suggests a potential connection between ASICs, mitochondrial dynamics, and energetic homeostasis in neurons (Savic Azoulay et al. 2020), underscoring the critical role of these channels in the maintenance of cellular integrity and highlighting further their importance as a potential therapeutic target.

### ASICs and neural plasticity in CNS

In the CNS, focus is given to ASIC1a, which is way more critical than ASIC2a or ASIC2b: because Ca^2+^-permeable, they directly drive synaptic plasticity and neuronal death in acidosis, while ASIC2 subunits are mainly modulators or helpers in assembly (Fuller et al. 2023).

**Fig. 2:**
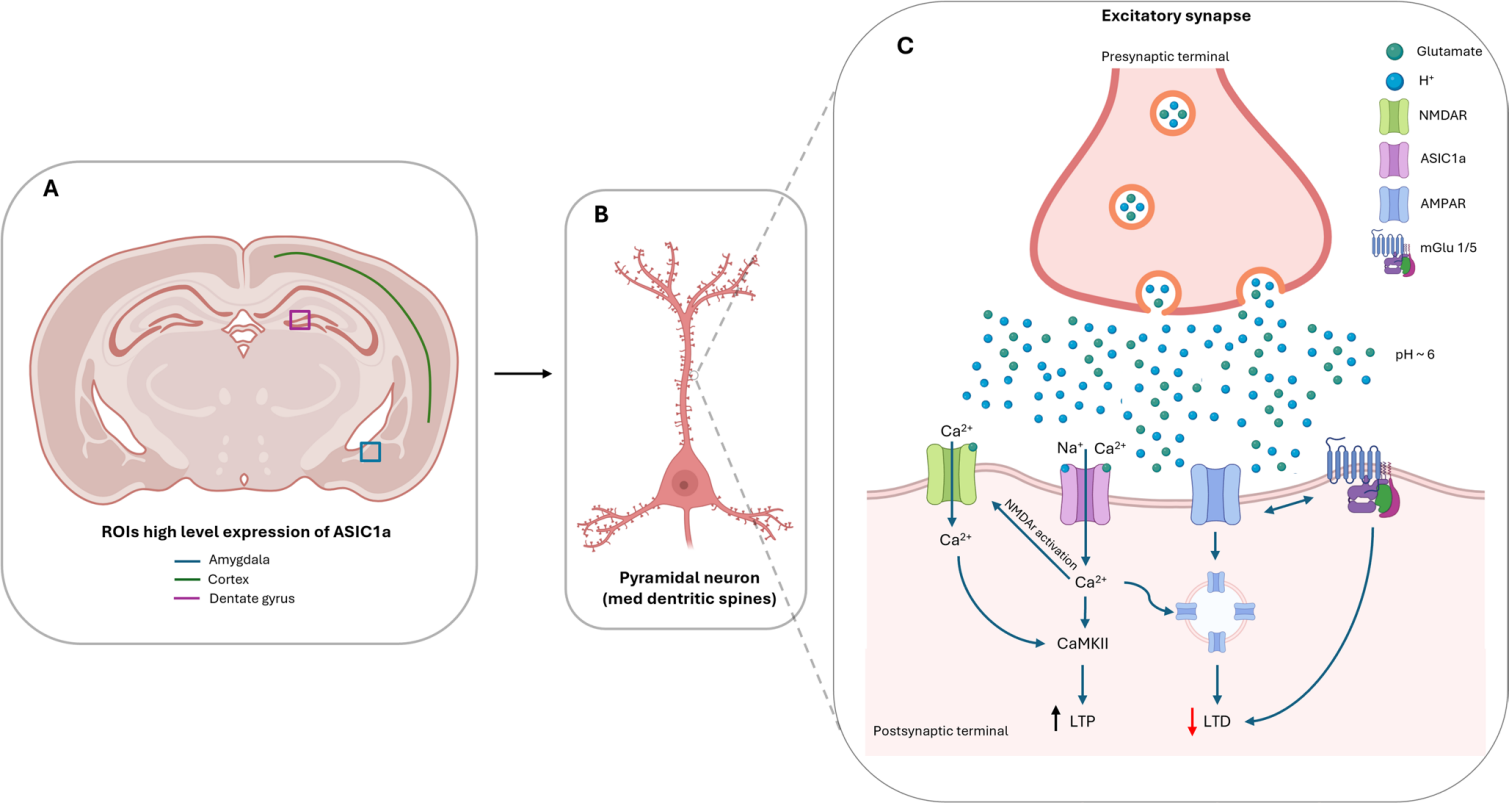
ASICs in Central Nervous system (CNS): **A** Representative regions of high ASIC1a expression in the CNS, including the amygdala, cortex, and dentate gyrus. ASIC1a is also broadly expressed across additional brain regions and in the spinal cord. **B** Schematic representation of a pyramidal neuron displaying dendritic spines, where ASIC1a channels are enriched and contribute to synaptic signalling. **C** Schematic representation of excitatory synapse, showing the role of ASIC1a in synaptic transmission and plasticity. ASCI1a activated by H^+^ contributes to Na^+^ and Ca^2+^ inflow. As a result of this, NMDARs and voltage-gated Ca^2+^ channels may be activated, triggering Ca^2+^-dependent signalling cascades, such as CaMKII activation. In these processes, metabotropic glutamate receptors (mGlu1/5) may also be modulated. Consequently, all these events end up influencing the two forms of synaptic plasticity: LTD, which involves AMPAR internalization, and LTP. (Created by Biorender.com)

Structurally, ASIC1a is enriched in the postsynaptic membrane, particularly at the level of the cell body, dendrites, and dendritic spines, mostly in excitatory synaptic areas, suggesting its influence on the synaptic plasticity process (Wemmie et al. 2002; Liu et al. 2016). Indeed, ASICs are required for long-term potentiation (LTP) in different brain regions (Fig. 2), such as the cortico-basal lateral amygdala synapses, which play a critical role in associative fear learning and memory (Du et al. 2014), and the hippocampus, where genetic deletion or pharmacological inhibition of ASIC1a does not completely abolish the LTP induction in the CA1 region (Liu et al. 2016). As mentioned earlier, activation of postsynaptic ASIC1a, by changes in extracellular pH, allows the influx of Na⁺ and, in some cases, Ca²⁺ ions, into postsynaptic terminals leading to membrane depolarization (Fig. 2) (Wemmie et al. 2002; Yermolaieva et al. 2004; Gao et al. 2005, 2015). The resulting depolarization and Ca²⁺ signaling promote the phosphorylation of CaMKII and facilitate the activation of NMDARs, thereby contributing to neuronal plasticity mechanisms (Gao et al. 2005, 2015). This process impacts synaptic transmission and neuronal excitability, ultimately contributing to long-term synaptic plasticity, particularly in the mechanisms of LTP and long-term depression (LTD). Re-establishing ASIC1a expression in the hippocampus fully rescued LTP in ASIC1a-deficient mice, while treatments with D-cycloserine (DCS), an NMDAR co-agonist, or using low Mg²⁺ concentration in artificial cerebrospinal fluid (aCSF) only partially restored LTP (Liu et al. 2016). Interestingly, reducing Mg²⁺ concentration did not hinder LTP induction, suggesting that ASIC1a influences hippocampal synaptic plasticity through both NMDAR-dependent and independent mechanisms (Gao et al. 2005, 2015; Buta et al. 2015). Notably, behavioral studies indicate that disrupting or overexpressing ASIC1a in mice alters learning and conditioning, proposing a functional interplay between ASIC1a and synaptic plasticity pathways. To further support this hypothesis, ASIC1a involvement has been shown in other studies where in ASIC1a knockout mice with a selective deletion of ASIC1a in GABAergic cells, including amygdala output neurons, completely abolished LTP in these cells and diminished fear learning to the same extent, as observed with the selective removal of ASIC1a in basolateral amygdala glutamatergic neurons (Chiang et al. 2015). Consequently, fear learning relies on ASICs-dependent LTP at various amygdala synapses, including cortico-basolateral amygdala input synapses and intra-amygdala synapses on output neurons (Chiang et al. 2015).

Given the role of ASIC1a in regulating synaptic plasticity, it is likely that they also influence LTD mechanisms, another crucial form of synaptic plasticity in the brain (Li et al. 2016; Mango and Nisticò 2019). However, their involvement in LTD was initially unclear and highly debated (Wemmie et al. 2002; Liu et al. 2016), but it has been shown that ASIC1a is involved in mGlu receptor-dependent LTD (mGluR-LTD) with a role in α-amino-3-hydroxy-5-methyl-4-isoxazolepropionic acid receptor (AMPAR) mediated transmission in CA1 pyramidal neurons of early adult mice (Mango et al. 2017), suggesting a post-synaptic mechanism of mGlu-LTD expression in mature spines (Kreple et al. 2014). Particularly, it has been evaluated that the subunit ASIC1a is involved in AMPAR internalization during mGlu-LTD, and ASICs inhibition reduces AMPAR phosphorylation induced by mGlu-LTD (Fig. 2) (Mango et al. 2017). This finding leads to more accurate and differentiated results compared to previous studies that could not explain the role of ASICs in LTD mechanisms. The involvement of ASICs in LTD is further supported by studies showing their contribution to NMDA-dependent LTD in adult mice, demonstrated with the use of selective and non-selective ASIC1a blockers such as Psalmotoxin-1 (PcTx1) and amiloride, while monitoring ASIC1a-mediated currents (Mango and Nisticò 2019). As in LTP mechanisms, the opening of ASIC1a may help depolarize the postsynaptic compartment, facilitating the influx of Ca²⁺ into the NMDAR within the dendritic spine, and initiating LTD (Mango and Nisticò 2019). Further supporting this, studies in ischemic brain models have revealed that ASIC1a activation enhances NMDAR currents, specifically through the GluN2B subunit (Ma et al. 2019), highlighting its potential role in modulating excitatory neurotransmission under pathological conditions. All together, these findings highlight the central role of ASICs in glutamatergic neurotransmission and synaptic plasticity, paving the way for new investigations and, more importantly, the design of novel therapeutics for neurodegenerative diseases characterized by dysfunctions in these pathways.

**Fig. 3:**
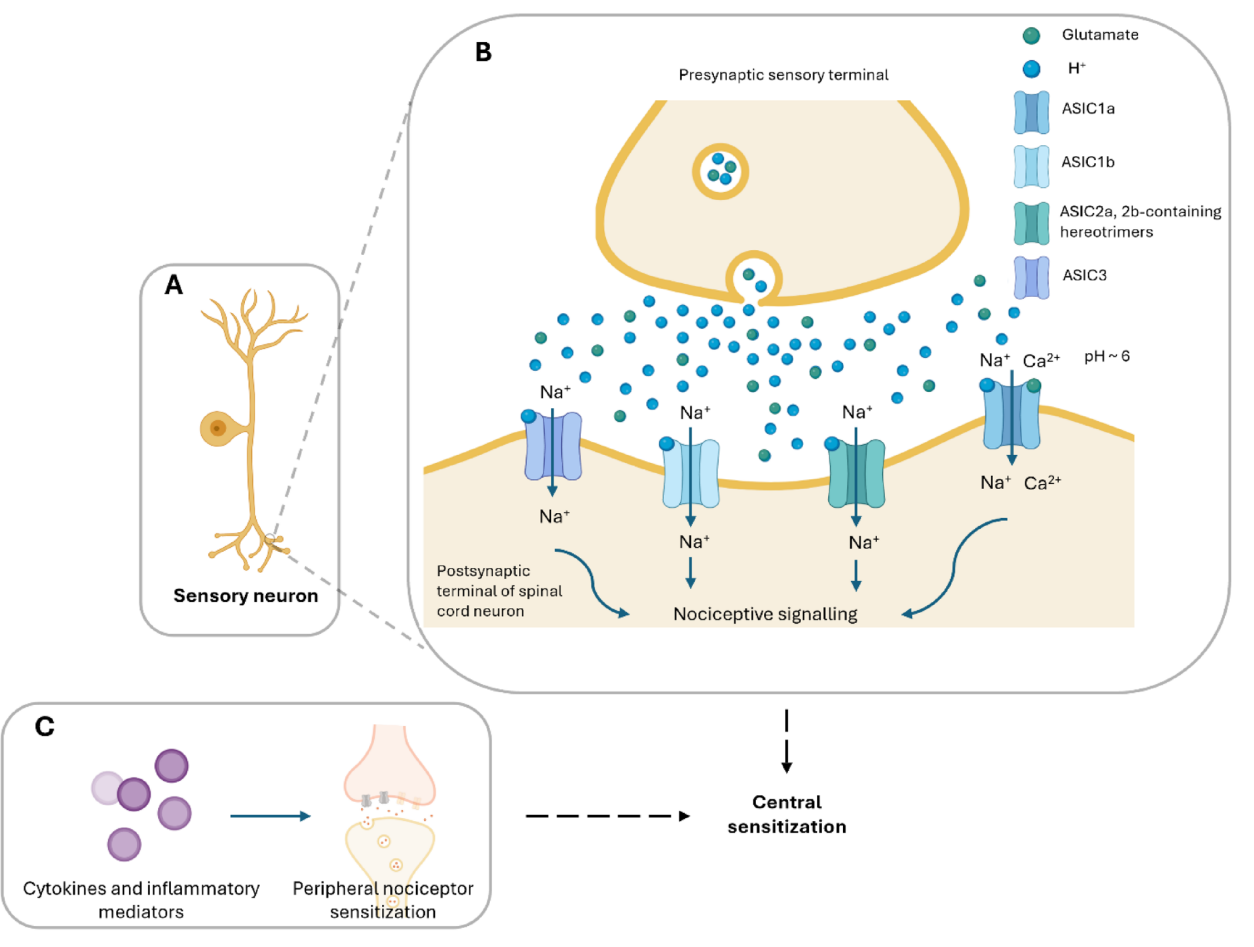
ASICs in peripheral nervous system (PNS): **A** A schematic diagram of a sensory neuron, showing peripheral nociceptor terminals sensitive to extracellular acidosis and their projection to the spinal cord. **B** A detailed view of the central terminal of a primary sensory neuron, which forms a synapse in the spinal cord’s dorsal horn. Under tissue acidosis, extracellular H⁺ ions activate acid-sensing ion channels composed of various subunits, i.e., ASIC1a, ASIC1b, ASIC3, and channels containing the ASIC2b subunit. Activation of these channels is mainly responsible for Na⁺ influx, resulting in depolarization of the neuron and enhanced nociception. ASIC1a channels can also allow limited Ca²⁺ permeability. Peripheral inflammation, which stems from the release of cytokines and other inflammatory substances, causes the sensitization of peripheral nociceptor terminals. This sensitization then amplifies the afferent signaling to the spinal cord, potentially resulting in central sensitization and the development of chronic pain. (Created by Biorender.com)

### ASICs across the PNS, their role, and the crosstalk with the CNS

The role of ASIC1a in synaptic plasticity is further reinforced by evidence that ASIC1a interacts with NMDARs and AMPARs to modulate excitatory postsynaptic currents in the anterior cingulate cortex (ACC) (Li et al. 2019). Such interactions highlight a critical link between ASICs and mechanisms of pain perception.

In the PNS, some ASICs are highly expressed in sensory neurons, where they play a crucial role in nociception and have been involved in mechanosensation, and possibly in synaptic plasticity (Deval and Lingueglia 2015; Huang et al. 2015), fundamental processes that shape how the body detects, interprets, and responds to the pain (Fig. 3). Recent studies with data from single-cell RNA sequencing have further characterized the expression profiles of ASICs subunits in various populations of sensory neurons, indicating the possibility that the expression profiles might contribute to the specialized functions of the ASICs subunits in the processes of nociception and sensory functions. In parallel, data from human tissues, including the dorsal root ganglion (DRG) of humans, have also confirmed the expression of ASIC1 and ASIC3 in sensory neurons (Hughes et al. 2007; Papalampropoulou-Tsiridou et al. 2020, 2022).

**Fig. 4:**
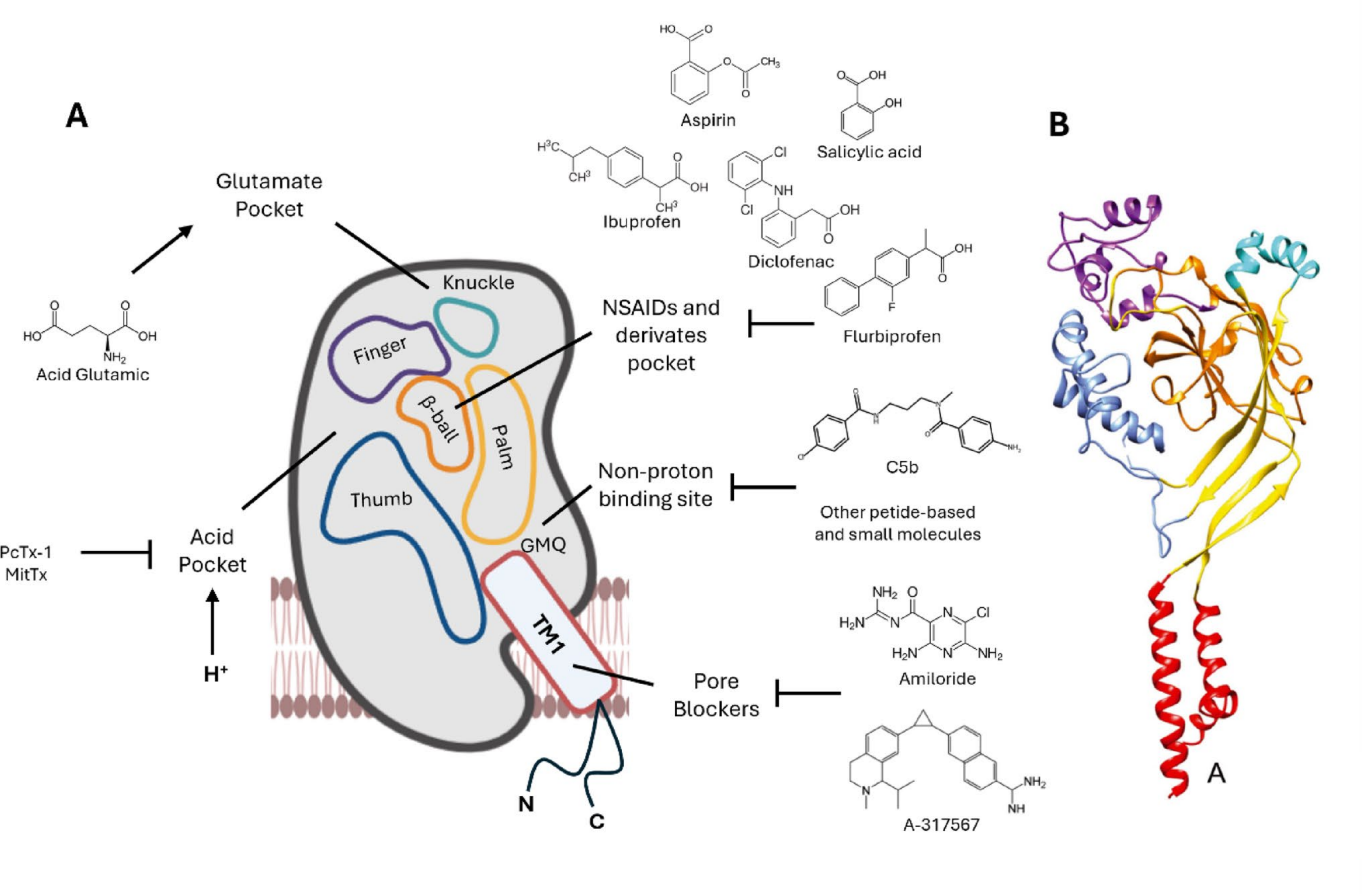
Structural organization and ligand binding of ASIC1a: **A** Schematic representation of ASIC1a structural domains, including the finger, knuckle, palm, β-ball, thumb and GMQ regions, as well as the membrane-associated TM1 helix. Several inhibitors and ligands are represented, each known to interact with distinct regions of the protein. **B** 3D ribbon structure of one subunit of ASIC1a, colour-coded to match the domain regions shown in the schematic illustration

In the PNS, ASIC1b and ASIC3 are predominantly expressed in the sensory neurons of the dorsal root ganglia (DRG), nodose ganglia, and trigeminal ganglia, making them key contributors to pain associated with tissue acidosis (Fig. 3) (Molliver et al. 2005; Deval et al. 2008; Ikeuchi et al. 2009; Walder et al. 2010).

Particularly, ASIC3 channels play an important role in nociceptive signalling at the interface between peripheral sensory neurons and spinal cord circuits, where their activation by acidosis can modulate pain transmission (Chafaï et al. 2023). By enhancing excitatory neurotransmission to central neurons, ASICs influence synaptic plasticity, driving LTP or LTD depending on the stimulus context and duration (Huang et al. 2015; Gobetto et al. 2021). This dynamic interplay underscores their role in shaping neural connectivity and signalling across both systems. Indeed, persistent ASIC3 activation in the PNS, such as in chronic pain conditions, can lead to long-term changes in CNS synaptic plasticity, manifesting as central sensitization, which involves enhanced synaptic responses in CNS pain pathways, changes in synaptic strength, receptor density, and neurotransmitter release, ultimately leading to persistent heightened pain sensitivity (Chafaï et al. 2023). Despite the different ASICs being expressed, peripheral extracellular acidification activates ASICs and results in depolarization of the membranes. The depolarization may result in the activation of voltage-gated Ca²⁺ channels, thus allowing Ca²⁺ ions to enter the cells and initiate various signalling cascades, including CaMKII phosphorylation (Wang et al. 2018; Duzhyy et al. 2021). Unlike in the CNS, where ASICs play a role in synaptic transmission and plasticity, in the PNS, ASICs mediate peripheral sensitization and transduction.

Modulation of ASIC3 function has the potential to reduce hyperexcitability of nociceptors in the periphery and limit the excessive sensory input that contributes to central sensitization and chronic pain. From this perspective, ASIC3 can be used to reduce the symptoms of peripheral neuropathies and other pain conditions (Chen et al. 2011; Wu et al. 2019; Kung et al. 2020). Furthermore, in the PNS, activating group I mGlu receptors in nociceptive DRG neurons can sensitize ASICs, which are implicated in acidosis-evoked pain (Gan et al. 2016). This effect may result from glutamate release during peripheral insults, including tissue acidosis, which can activate mGlu receptors and enhance ASIC signalling. Interestingly, glutamate has also been reported to potentiate ASIC-mediated currents, although this effect has been demonstrated primarily for ASIC1a (Lai et al. 2024a). However, these interactions have not yet been examined in integrated neuro-peripheral models, and their contribution to coordinated CNS–PNS plasticity remains to be fully established. Nevertheless, given the well-characterized role of mGluRs in regulating synaptic plasticity in central neurons, herein, modulation of mGluR signaling has been proposed as a potential indirect mechanism for influencing ASICs function. In disorders characterized by coupled peripheral and central sensitization, such an approach may offer a conceptual framework for targeting maladaptive plasticity across interconnected neural circuits, rather than acting on peripheral or central components in isolation.

## ASICs in neurological disorders as an innovative pharmacological target

All these findings highlight ASICs’ critical role in a range of neurological disorders, thus prompting the possibility of targeting these channels to mitigate disease progression. However, it is that all the potential ASICs modulators interact with distinct channel subunits, and these unique interactions between modulatory molecules and subunit-specific sites could pave the way for novel drug discovery, as demonstrated in Fig. 4.

### Direct pharmacological targeting of ASICs

The main ASIC1a inhibitors are peptide-based compounds that represent a promising therapeutic strategy by preventing pathological role of the ASIC1a channel. Among them, we can find PcTx1 that exerts a protective effect in models of Amyotrophic Lateral Sclerosis (Wu et al. 2021), ischemia (Xiong et al. 2004; McCarthy et al. 2015), Parkinson’s disease (Gu et al. 2016), and Alzheimer’s disease (Mango and Nisticò 2018). The involvement of ASIC1a in synaptic plasticity deficits within CA1 pyramidal neurons in AD models was first demonstrated by Mango et al. (Mango et al. 2017), revealing that Aβ amplifies mGluR-dependent long-term depression (mGluR-LTD), leading to impaired synaptic function. Notably, PcTx1 counteracted this effect, restoring LTD to control levels. On the contrary, PcTx1’s high molecular weight and peptide structure restrict its oral bioavailability and ability to cross the blood-brain barrier (BBB) (Renukuntla et al. 2013). However, it is also true that under certain pathological conditions, such as stroke, or following pathological insult to the CNS, such as multiple sclerosis, BBB permeability can be transiently increased to the degree that it can be considered in some instances ‘leaky’. However, this BBB leakage is heterogeneous, unpredictable, and erratic, making it not a reliable route for drug delivery systems. These pharmacokinetic limitations highlight the need for novel and creative drug delivery strategies, or, at the very least, the production of smaller molecules that are protein-like and more stable and permeable, such as Hi1a or C5b. Hi1a, as the most potent ASIC1a inhibitor, has proven useful to validate ASIC1a as a target to further develop in stroke, myocardial infarction, and heart transplantation (Chassagnon et al. 2017; Redd et al. 2021, 2024). Indeed, C5b, a small PcTx1-based compound, has demonstrated efficacy in reducing infarct volume and improving neurological outcomes in ischemic models, providing hope for reducing the long-term impacts of ischemic brain injuries, such as those occurring during strokes (Qi et al. 2022).

In addition, APETx2 is another peptide isolated from sea anemone that can inhibit the ASIC3 current in rat sensory neurons (Diochot et al. 2004), while in vivo studies it can prevent the development of mechanical hypersensitivity in acid-induced muscle pain and also reverse mechanical hypersensitivity (Karczewski et al. 2010). However, it can also potentiate ASIC1b and ASIC2a currents at concentrations 30–100 times greater than the concentration of the inhibitory dose for ASIC3. Other peptide inhibitors are mambalgins, a three-finger toxin from the venom of black mamba that selectively blocks ASIC1-containing channels and causes analgesic effects in rodent models of pain without affecting the cardiovascular system (Diochot et al. 2012). Interestingly, natural peptides like dynorphin A and big dynorphin have been found to enhance ASIC1a currents (Sherwood and Askwith 2009; Leisle et al. 2021), showing just how complex the body’s own regulation of ASIC channels can be, and reinforcing their importance as targets for drug development. Small molecules, such as A-317567 (amiloride-based) (Dubé et al. 2005), represents an interesting approach for medicinal chemistry to achieve peripheral specificity, but it will require further optimization to ensure robust subtype selectivity, as the case of lidocaine, which specifically inhibits ASIC1a currents (Lin et al. 2011).

Unlike these peptide inhibitors, the peptide toxin MitTx from the Texas coral snake (Micrurus tener) is a strong ASIC1a agonist, and it activates ASIC1a at neutral pH, facilitating ASIC2a currents at acidic pH (Bohlen et al. 2011). In vivo, MitTx induces pronounced pain behaviors in mice through the activation of ASIC1a on sensory neurons and is therefore a useful tool for identifying ASIC-expressing nociceptors and evaluating potential analgesic therapies.

Another interesting chemical is memantine, an FDA-approved AD treatment known for its NMDAR-blocking properties, which has also been shown to modulate ASICs activity in a voltage- and pH-dependent manner, playing a role in both excitotoxicity and Ca²⁺ dysregulation (Tikhonova et al. 2015; Shteinikov et al. 2018). Although NMDAR therapies can lead to significant psychotic side effects, recent studies have highlighted the potential of ASIC1a in mitigating these conditions through its glutamate-binding pocket (Lai et al. 2024a). Specifically, it has been shown that glutamate binding to ASICs can enhance their affinity for H^+^, further exacerbating ischemic neurotoxicity (Lai et al. 2024b, a). Targeting this specific interaction, without impacting NMDAR function, emerges as a promising strategy to reduce ischemic injury while maintaining synaptic integrity, thereby safeguarding overall neuronal health (Lai et al. 2024b, a).

Also, Nerve growth factor (NGF) has been identified as an ASICs modulator in AD models. Normally, it mitigates Aβ-induced alterations by regulating amyloid precursor protein (APP) processing and preventing memory impairment (Triaca et al. 2021), but it has been found to enhance ASIC1a expression via the NF-κB pathway (Wei et al. 2020), suggesting that a combination of NGF and ASICs inhibitors could offer a synergistic therapeutic strategy.

Other inhibitors, such as the antidepressant mirtazapine, can modulate ASIC1 activity, offering dual benefits of pain relief and neuronal protection (Bektur et al. 2019), thus addressing both the physical and psychological burdens of Diabetic Peripheral Neuropathy (DPN), a debilitating complication of diabetes mellitus involving damage to peripheral nerves, aberrant synaptic plasticity, and heightened neuropathic pain (Pop-Busui et al. 2022). In the same context and somehow conversely given the different ASICs subtype, the pharmacological inhibition of ASIC3 protected mice from thermal hypersensitivity in a mouse model of lipid-rich diet induced obesity, which can lead to pre-diabetic state (Negm et al. 2024). Additionally, resveratrol, a SIRT1 activator, has been shown to inhibit ASIC-mediated currents in dorsal root ganglion neurons, suggesting a direct modulatory effect on acid-sensing ion channels, in addition to its known antioxidative and anti-inflammatory actions that influence ASIC3 expression in inflammatory conditions (Deval et al. 2008; Wei et al. 2022).

In the context of natural molecules, paeoniflorin also demonstrates neuroprotective effects by reducing ASIC1a-mediated Ca^2+^ influx and enhancing autophagy and protecting dopaminergic neurons (Cao et al. 2010), providing potentially an alternative therapeutic approach that could complement traditional pharmacological interventions in the field of PD. However, natural compounds might be characterized by pharmacokinetic problems, such as instability, limited solubility, short half-life, and interaction with other drugs. Despite these challenges, it is possible to overcome them with innovative strategies to deliver substance to both the peripheral and central systems (Dalamagkas et al. 2016; Furtado et al. 2018; Nance et al. 2021; Maisto and Mango 2024). Moreover, natural compounds continue to serve as a valuable foundation for developing new molecules in the field of drug development, natural-based molecules, potentially providing insights also for ASICs targeting molecules.

The promising use of ASIC1a as a therapeutic target has been demonstrated in different fields of interest, particularly in rare disease, such as Fabry disease (FD), characterized by accumulation of glycosphingolipids in lysosomes leading complications in kidney, heart and nervous system (Bolsover et al. 2014): in an in vitro FD model, ASIC1a has been found overexpressed, resulting in a phosphorylation of the ERK/MAPK pathway, which may contribute to symptomatic pain, and ASIC1a inhibition with PcTx1 has blocked this pathway activation (Castellanos et al. 2020), suggesting a new therapeutic approach for FD.

Similarly, recent studies have also provided new insights focusing on ASICs channels in tumor treatments, given that the local pH value of malignant tumor tissues is between 6.0 and 6.9 (Wang et al. 2022). It has been shown that ASIC1 expression in glioma cell lines, like U87MG, boosts cell migration in response to weak acidic conditions (Sheng et al. 2021). Inhibiting or knocking down ASIC1 significantly reduces this migration, highlighting its role in the process. On the other hand, increasing the amount of ASIC2 expression appears to suppress glioblastoma cell migration and proliferation (Vila-Carriles et al. 2006), suggesting that targeting ASIC1 could be a promising strategy for glioma therapy, avoiding the side effects of the conventional treatments.

Finally, the gene delivery therapy, silencing ASICs overexpression, represents a promising approach for managing neuropathic pain and preventing excitotoxicity and for validating ASIC3 as a key mediator of nociception (Sluka et al. 2007).

For example, Herpes Simplex Virus (HSV)–based vectors, which efficiently target sensory neurons, have been used to re-express ASIC3 in dorsal root ganglia, restoring pain sensitivity; conversely, ASIC3 silencing via shRNA or miRNA was able to reduce hyperalgesia, highlighting its therapeutic potential (Deval et al. 2008; Walder et al. 2011). However, it is important to acknowledge the challenges posed by pharmacokinetics, including the side effects associated with gene delivery, but also species differences and limited mechanistic data, which limit clinical translation. In this context, exploring innovations in drug delivery systems and developing analogues with enhanced bioavailability could further expand the therapeutic potential of ASIC-targeting compounds, particularly peptide toxins whose clinical translation is limited by stability and blood–brain barrier permeability (Zhou et al. 2021).

Moreover, the passage from preclinical to clinical research must consider human genetics associated with the drug target: across therapeutic areas, genetic support linking a drug target to a disease increases the likelihood of success in terms of the clinical outcome (King et al. 2019). This would mean that the variation previously observed in ASIC1 would be more relevant to ischemic stroke and coronary heart disease (Xiong et al. 2004; Redd et al. 2021), or panic disorder and related amygdala phenotypes connected to anxiety (Wemmie et al. 2006; Smoller et al. 2014; Quagliato et al. 2018). There are also emerging summaries suggesting tentative associations for ASIC2 with developing depressive and anxiety phenotypes, but not as clearly across varying cohorts (Smoller et al. 2014; Gugliandolo et al. 2016; Fuller et al. 2023). Conversely, new published human case control studies have not encouraged associations for any of the selected ASIC3 SNPs in fibromyalgia, despite a solid preclinical pain biology (Zontul et al. 2024). Overall, although cardiovascular and ischemic phenotypes have been observed, and some psychiatric signals seem more artificial, it is important to note that there have been no robust genome-wide human genetic associations identified for ASIC subtypes in neuropsychiatric or sensory disorders (Li et al. 2023; Adams et al. 2025). This represents a missed opportunity for targeted research and therapeutic development. These findings, combined with functional studies in animal models, support the view that ASICs are key modulators of neuronal function and represent compelling targets for therapeutic intervention in various neuropsychiatric and sensory disorders.

### Repurposed drugs and endogenous pathways influencing ASICs: therapeutic perspectives

Indirect modulation of ASICs also offers an innovative approach to managing disorders linked to these channels by targeting upstream pathways, structural interactions, and systemic mechanisms, rather than directly inhibiting the channels themselves, helping to minimize the risk of adverse effects. A prime example is lichexanthone, a natural xanthone derivative, which has demonstrated significant pain-reducing effects in models of ASIC-mediated hyperalgesia, such as acidic saline-induced pain (Ribeiro Liberato et al. 2024). Notably, its effects are reversed by amiloride, which is consistent with a potential involvement of ASIC-related pathways, although this observation should be interpreted with caution given the non-selective nature of amiloride (Ribeiro Liberato et al. 2024).

Other promising indirect modulators include serine protease inhibitors such as nafamostat, sepimostat, and diminazen, which inhibit ASICs’ activity via a voltage-dependent mechanism, preventing ion flow through the channel pore under acidic conditions (Zhigulin et al. 2023). These compounds alter the structural and functional dynamics of the channels, offering potential pharmacological applications for inflammatory pain, ischemic stroke, and other ASIC-associated disorders. Specifically, nafamostat, with its established clinical safety profile, further underscores the promise of these agents for human therapeutic use (Zhigulin et al. 2023).

In line with this, also non-steroidal anti-inflammatory drugs (NSAIDs), such as ibuprofen, not only exert their primary effects through cyclooxygenase inhibition but also modulate these channels, particularly by inhibiting ASIC1a currents (Voilley et al. 2001). This dual mechanism of action enhances the effectiveness of ibuprofen in treating conditions where ASICs dysregulation contributes to neuroinflammation and nociceptive pain. Notably, ibuprofen’s voltage-independent inhibition of ASIC1a indicates that it interacts with an extracellular binding site, expanding its therapeutic potential for disorders associated with ASICs dysfunction (Fechner et al. 2021).

Recently, new molecules based on pyrazoles were able to modulate the activation of nociceptors (Bencheva et al. 2019) or brain ischemic insult (Gornati et al. 2021), with the molecular targets being the ASIC1a channels, opening new insights in this field for drug discovery.

Similarly, beyond pharmacological agents, endogenous molecules such as neuropeptides, cytokines, histamine, lactate and lipids also modulate ASICs activity (Allen and Attwell 2002; Smith et al. 2007; Malcher-Lopes and Buzzi 2009; Marra et al. 2016; Barygin et al. 2017; Vyvers et al. 2018; Azoulay et al. 2022; Castellanos et al. 2024). These molecules can either enhance or inhibit the channel’s function depending on the physiological context. For instance, histamine potentiates ASICs activity (Nagaeva et al. 2016), while lactate contributes to its desensitization (Azoulay et al. 2022), specifically potentiating ASIC1a currents (Immke and McCleskey 2001; Allen and Attwell 2002). Understanding these natural regulatory mechanisms provides valuable insights for developing pharmacological agents that can modulate ASICs activity in a targeted manner, as well as revealing interesting interplays between these channels and other neuronal components.

Collectively, these strategies highlight the therapeutic potential of targeting ASICs through indirect mechanisms. The approach offers several advantages, including improved specificity, reduced side effects, and the ability to modulate ASICs within broader systemic contexts. While this shift towards indirect modulation represents a significant advancement in therapeutic innovation, offering new possibilities for managing ASIC-related disorders, future research should focus on refining these approaches, exploring combinations of indirect modulators, even for prevention, and assessing their long-term safety and efficacy, as the exact contribution of ASICs modulation to therapeutic outcomes is often unclear, and off-target effects, pharmacokinetics, and systemic context must be considered.

## ASICs beyond neurons: roles in glial cells

The role of ASICs in neuronal signalling has long been studied, yet their involvement in glial cells, particularly astrocytes, microglia, and oligodendrocytes, remains an area of growing interest. These glial cells, essential to both normal brain functions and the progression of neurological diseases (Goshen et al. 2007; Santello et al. 2019; Wright-Jin and Gutmann 2019; Umpierre and Wu 2021), have begun to reveal a complex relationship with ASICs, also given their involvement in Ca^2+^ signalling and in regulating key glial functions such as cytokine release and glutamate uptake. Indeed, cytokines such as IL-1β, TNF-α, and IL-6 have been studied in connection with ASIC1a channels, particularly acting on their redistribution given by acidic environment (Castellanos et al. 2024).

Notably, in recent reviews have highlighted some essential context to this naive field. Foster et al. (2021) (Foster et al. 2021) stated that the ASICs are expressed in resident glial cells as well as in infiltrating immune cells of the CNS where they play roles in neuroinflammation and immune regulation. In particular, these channels may modulate cytokine release from glial cells, shaping the inflammatory environment. Whereas, Cegielski et al. (2022) (Cegielski et al. 2022) focused on glia, and included evidence of ASICs subtype expression across astrocytes, microglia, and oligodendrocytes and their involvement in synaptic modulation, demyelination, inflammation and neurodegeneration. Together, these reviews provide context for considering ASICs not only in an individual subtype of glial cells but in the larger context of neuroimmunology. Importantly, these reviews also highlight critical shortcomings in knowledge, most notably the critical need for cell-type-specific and mechanistic studies to confirm the functional roles of ASICs in glial physiology and disease.

### Astrocytes

Studies have shown that ASIC1a, ASIC2a, and ASIC3 are expressed in astrocytes, with ASIC1a being the most prominent isoform (Huang et al. 2010). Interestingly, ASICs in astrocytes exhibit a unique pattern of localization, predominantly found within the nucleus, in contrast to their neuronal counterparts, which are mainly localized to the plasma membrane and cytoplasm (Huang et al. 2010). This differential subcellular distribution suggests that ASICs in astrocytes may exert distinct functional roles, potentially influencing their contribution to neuroinflammation, neuronal support, and overall brain homeostasis. In particular, ASIC activity in astrocytes may regulate glutamate uptake, thereby modulating excitotoxicity and synaptic transmission, as well as cytokine release during inflammatory conditions. In this context, during epilepsy, an abnormally high ASIC1a expression has been found within astrocytes, prompting ASIC-astrocytes interplay in cognitive function impairment (Li et al. 2025). Alongside ASICs, transient receptor potential vanilloid type 1 (TRPV1) channels are also present in astrocytes, primarily localized to the cytoplasm and membrane, particularly in rat spinal dorsal horn astrocytes (Tóth et al. 2005; Huang et al. 2010). The ASICs’ role is given by eliciting two distinct ionic currents upon acidic stimulation in cultured rat astrocytes: a transient current mediated by ASICs and a sustained current mediated by TRPV1 (Huang et al. 2010). This dual response suggests that ASICs and TRPV1 channels may contribute to astrocytic signalling in complementary yet distinct ways, potentially influencing their roles in neuroinflammation, neuronal support, and synaptic plasticity.

### Microglia

Microglia also express ASIC1a, ASIC2a, and ASIC3, which mediate inflammatory responses and are activated in conditions like neurodegeneration and ischemia (Cegielski et al. 2022). Notably, studies on cultured rat microglial cells have shown that inhibiting ASICs with PcTx1 and amiloride provides negative feedback, leading to a reduction in cytokine levels and subsequently dampening inflammation (Yu et al. 2015) as well as microglial phagocytosis in a multiple sclerosis model (Vergo et al. 2011; Ortega-Ramírez et al. 2017). These findings highlight a direct functional role for ASICs in regulating microglial cytokine release, potentially influencing both local and systemic inflammatory responses. Furthermore, the interplay between microglia and ASICs channels has been observed indirectly, particularly in the spinal cord, where microglia activation, induced by morphine treatment, has been regulated by ASIC3 inhibition (Gei et al. 2024), suggesting their potential use in preventing morphine tolerance. Indeed, it has been demonstrated that microglial activation and inflammatory responses are significantly modulated by ASICs in animal models (Ortega-Ramírez et al. 2017), shedding light on their role in contributing to axonal degeneration in autoimmune inflammation, such as multiple sclerosis (Friese et al. 2007). Despite the promising potential of ASICs in microglial activation, direct evidence remains sparse. A key example is the modulation of the NLRP3 inflammasome, a critical driver of neuroinflammation in microglia (Xu et al. 2025). Specifically, inhibition of ASICs has been shown to reduce NLRP3 activation in different animal models (Zhao et al. 2021; Yang et al. 2023), proposing that ASICs may play a role in regulating microglial-mediated inflammation. However, further investigation is crucial to fully understand the direct connection between ASICs activity and inflammasome regulation in microglial cells, to better understand their potential as a new therapeutic target. Overall, given their role in excitotoxic environments, future studies should also explore whether ASICs influence glutamate clearance in microglia.

### Oligodendrocytes

Oligodendrocytes, which are critical for myelin sheath formation and axonal health, on the other hand, demonstrate ASIC-like responses when exposed to an acidic extracellular medium (Waldmann and Lazdunski 1998). At the transcriptome level, these cells express ASIC1a ASIC2 and ASIC4, while there is no evidence of ASIC1b, and ASIC3 was only weakly measured (Feldman et al. 2008). Notably, the evidence for ASIC2 in cultured oligodendrocytes arises from the use of a non-selective primer set that could not distinguish between variants ASIC2a and ASIC2b. In sharp contrast, when primers specific to the variants were used on adult rat white matter, ASIC2a, but not the subtype 2b, was detected alongside ASIC1a (Feldman et al. 2008). However, protein evidence has demonstrated ASIC1a in white matter oligodendrocytes in the form of immunofluorescence, but there is no Western blot or protein expression data for the ASIC2 splice variants. Overall, these findings suggest that ASIC1a is the most consistently documented subtype in oligodendrocytes, with some additional but overly cautious evidence for ASIC2 expression (Feldman et al. 2008). Despite the different ASICs gene expression, under whole-cell recording conditions, the majority of detectable functional ASICs seem to be contributed by the ASIC1a subtype (Feldman et al. 2008), causing membrane depolarization and elevating intracellular Ca^2+^, potentially synergizing with AMPAR, NMDAR, and voltage-gated channels, and possibly influencing glutamate uptake and excitotoxic vulnerability, a connection that needs to be further investigated. Taken together, these findings lead to the hypothesis that channels containing ASIC1a might be involved in Ca²⁺-dependent signaling in oligodendrocytes and thus might play a role in excitotoxic cell death susceptibility and in myelin maintenance and repair. Nevertheless, there is a paucity of knowledge about a potential direct role of ASIC1a in oligodendrocyte death and regeneration.

However, the association of a clinical benefit to the ASIC1a manipulation represents a challenge, as confirmed in the study of ASIC1a as a potential therapeutic target in primary progressive multiple sclerosis that arose from a clinical trial using amiloride, a diuretic that has been shown to inhibit ASIC1a: after an initial evidence for a neuroprotective effect, when a follow-up study was conducted to investigate a potential benefit, no benefit could be confirmed (Arun et al. 2013).

Taken together, ASICs expression and activity in glia are extremely specific for glia type: although signalling involving ASICs occurs in astroglia, microglia, and oligodendroglia, the quality and intensity of evidence significantly differ for each type. Additionally, in many instances, the observed effects seem to be indirect, either through modulation of Ca²⁺ signalling, or through involvement in the inflammatory pathways, or through neuron–glia interactions, even though there is growing evidence that ASICs directly influence glial functions such as cytokine release and glutamate uptake, which may contribute to disease progression in a condition- and stage-specific manner. This complexity underlines both the promising potential and challenge associated with glial ASICs in neurological disorders.

Another point is that glial ASICs are likely to contribute to or influence disease development in a manner that is both condition-specific and stage-specific, highlighting the need to conduct future studies with cell type-specific methodologies as well as improved pharmacological interventions to distinguish between primary causality and secondary reactions to glial ASICs.

## Conclusions

ASICs are gaining attention as critical modulators across both CNS and PNS. This review will contribute to providing a full perspective of the role of ASICs in a wide range of neuro-disorders, by considering the therapeutic possibilities of ASICs modulation, both in terms of synaptic plasticity in the CNS as well as pain and sensory dysfunctions in the PNS, and potential relevance in diseases involving both systems. Overall, the evidence suggests that ASIC1a and ASIC3 represent the most viable therapeutic targets to be modulated, especially in neurodegenerative disorders (e.g. Alzheimer’s disease, Parkinson’s disease, ischemia), to provide neuroprotective effects. While pharmacological strategies targeting the channels are progressing, there are several small molecules and peptide-based inhibitors identified across multiple pathological conditions that could affect the modulation of the ASICs. However, a drug targeting ASIC1a in the brain might produce very different pharmacology effects than one targeting ASIC1b or ASIC3 in peripheral sensory neurons, since ASICs subtypes are not expressed uniformly in CNS and PNS, and pharmacological tools currently available are not strongly subtype selective. Therefore, there are still barriers that need to be addressed before translational success, such as implementing drug discovery and delivery to the target site while avoiding off-target effects. In line with this, future work should involve the development of selective ASICs modulator compounds, by testing their therapeutic efficacy in preclinical models and potentially translating it to the clinic. In conclusion, ASICs have real relevance as a potential and effective target for new drug development and discovery, considering their impact on multiple neuro-disorders.

## Data Availability

All the data reported in this review were found in literature through PubMed and Google Scholar sources.
